# High-Resolution Spatial Surveillance of Sexually Transmitted Disease Risk in Urban Japan Using Hierarchical Hexagonal Grids (H3) and Quantum Geographic Information System (QGIS)

**DOI:** 10.7759/cureus.94061

**Published:** 2025-10-07

**Authors:** Ryo Horiike, Wakana Kitsuda, Natsuki Nagai, Megumi Sato, Izumi Kondo, Motoyo Nawate, Harumi Bando

**Affiliations:** 1 Nursing, Nara Medical University, Kashihara, JPN; 2 Nursing, Osaka Medical and Pharmaceutical University Hospital, Takatsuki, JPN; 3 Health and Longevity Promotion Division, Fushimi Ward Office Health and Welfare Center, Kyoto, JPN; 4 Regional Health Division, Osaka Prefecture Izumi Health Center, Izumi, JPN

**Keywords:** geographic information systems, public health nursing, qgis, sexually transmitted diseases, spatial analysis

## Abstract

Introduction

Urgent action is required to combat sexually transmitted diseases (STDs), such as syphilis. This study aimed to examine whether the combination of hierarchical hexagonal grids (H3) (Uber Technologies, Inc., San Francisco, CA, USA) and Quantum Geographic Information System (QGIS) (OSGeo, Grut, Switzerland) can capture localized clustering and short-term fluctuations of estimated STD-related risk behaviors with high spatial resolution while preserving anonymity, and to evaluate its potential utility as a public infectious disease surveillance method.

Methods

A field survey was conducted in District A, an urban area in Japan (80 m × 60 m), between July 16 and 27, 2024, from 6:30 to 8:00 p.m. Estimated female commercial sex workers (CSWs) and involved males were recorded using a GPS application, collecting only date, time, latitude-longitude, and gender. The data were processed in QGIS and aggregated at H3 resolutions 13 (43.9 m²) and 14 (6.3 m²). We also assessed fluctuations in the data during an incidental police intervention that occurred during the observation period. Maps displayed anonymized building footprints, and no contact with individuals or collection of personal information occurred.

Results

At H3 resolution 14, clusters of up to four observations were detected in cells near the central intersection, while resolution 13 allowed recognition of broader spatial patterns. Over the 10-day observation period, a total of 96 observations were recorded. Spatial clustering varied by time period and gender. An external factor (police intervention) resulted in a temporary reduction of approximately 70% in observations, followed by a rapid recovery, which was visualized on the grid.

Conclusions

High-resolution hexagonal grid analysis using H3 and QGIS provides a practical method to monitor the spatial dynamics of STD-related risk behaviors without handling personal information. Its independence from administrative boundaries enhances generalizability, enabling flexible application in response to infection trends and supporting evidence-based public health nursing activities in infectious disease surveillance.

## Introduction

Reports of sexually transmitted diseases (STDs) in Japan have risen markedly in recent years; in 2024, syphilis notifications exceeded 14,000 cases, reaching a historical peak [1]. In the same year, more than 50% of male cases were in their 20s-50s, and more than 50% of female cases were in their 20s-30s [1]. Moreover, within the preceding six months, 35.5% of women reported experience with commercial sex work (CSW) and 40.9% of men reported use of such services, suggesting that the sex industry may constitute a major route of transmission [1]. Although inadequate preventive practices in sex-industry settings may contribute to STD spread, detailed measurement of these risk behaviors is challenging due to social stigma and privacy concerns. In addition, while Japan’s National Epidemiological Surveillance of Infectious Diseases collects physician reports to local public health centers, these notifications alone are insufficient to delineate geographic transmission pathways [2].

In spatial epidemiology, an open-source hierarchical hexagonal grid system (H3) (Uber Technologies, Inc., San Francisco, California, USA) has gained attention [3,4]. H3 enables continuous partitioning of space at multiple resolutions and provides analysis units that are homogeneous and independent of administrative boundaries [4]. During the COVID-19 era, high-resolution clustering and mobility-based analyses were reported, and applications have been described for diseases such as tuberculosis and COVID-19 [5,6]. These studies highlight the value of high-resolution grids for characterizing respiratory infection dynamics. However, to the best of our knowledge, applications of H3-based spatial surveillance for STDs have not been reported in the peer-reviewed literature.

Because STD transmission depends on face-to-face contacts and behaviors that are highly localized and variable over short time scales, conventional administrative units may fail to capture spatial clustering adequately. Research that handles individual location information also requires methods that allow fine-grained analysis while maintaining privacy protections [7]. By aggregating point locations into hexagonal cells, H3 can de-identify coordinates and visualize spatiotemporal patterns without handling personal identifiers, making it a plausible approach for STD surveillance.

This study primarily aimed to determine whether the combination of H3 and the open-source geographic information system Quantum Geographic Information System (QGIS) (OSGeo, Grut, Switzerland) can capture localized clustering and short-term fluctuations in estimated STD-related risk behaviors at high spatial resolution while preserving anonymity.

As secondary objectives, we explored the potential utility of this approach for public infectious disease surveillance and assessed whether it could visually reflect behavioral changes following external events, such as police intervention. Field-collected global positioning system (GPS) data were aggregated to H3 resolutions 13 and 14 to evaluate whether this method offers a practical, scalable, and privacy-preserving framework for spatial monitoring of STD-related behaviors.

## Materials and methods

Study area

**Figure 1:**
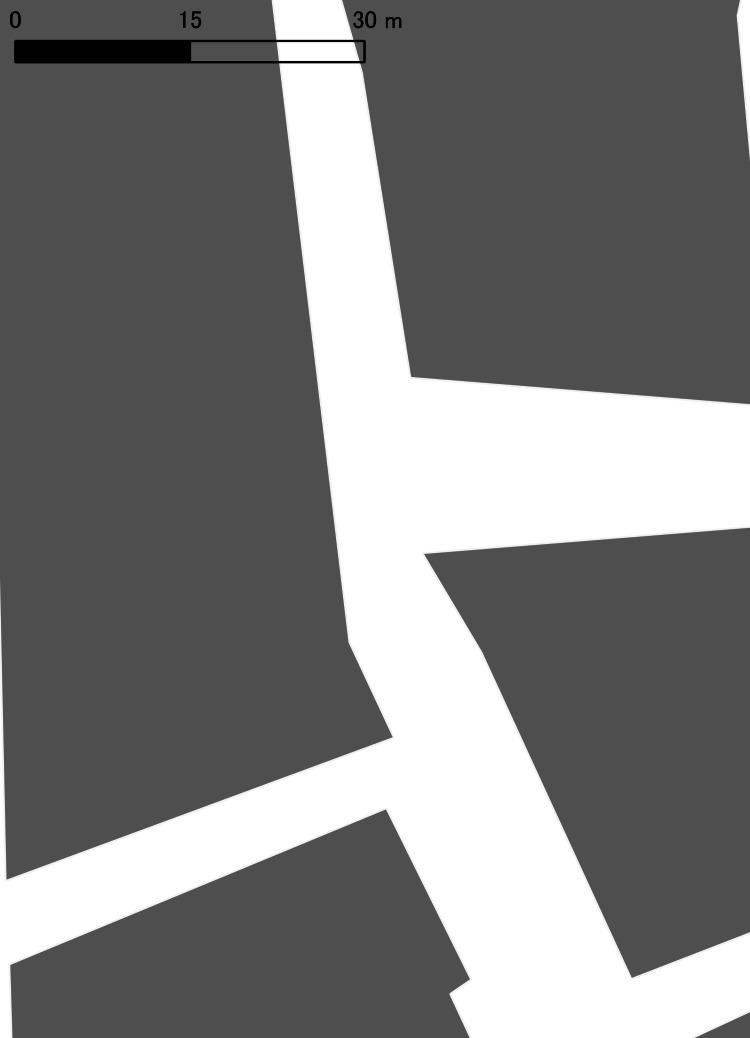
Building footprints in District A Gray polygons indicate building footprints; white areas represent roads. © OpenStreetMap contributors.

Field observations were conducted in District A, located within an urban area in Japan. The site has previously been subject to police crackdowns on street-based prostitution, and posters indicating the illegality of street prostitution were displayed by the police. The target area was a rectangular block (approximately 80 m × 60 m) bounded by pedestrian-only streets (road widths 4-10 m), consisting of a north-south through street and branching east-west streets. A central intersection was present within the block (Figure 1). Multiple love hotels of five stories or higher were concentrated in the area. After 6:00 p.m., pedestrian traffic by local residents was sparse, and women presumed to be commercial sex workers (CSWs) were consistently observed standing on the street.

To avoid privacy concerns and stigmatization of the neighborhood, building footprints from OpenStreetMap were traced in QGIS with the following modifications before publication: (1) orientation was altered, (2) building shapes were adjusted, and (3) street and business names were removed; precise coordinates are not reported in the manuscript. These steps were taken to preserve spatial relationships while preventing location identification [8].

Study population

We defined the study targets as follows:

(1) Women who remained standing on the sidewalk, engaged in conversation with a nearby male, or used a smartphone, and then walked in the same direction as the male were operationally classified as CSWs. Women merely passing through the area without stopping, as well as delivery personnel, were excluded.

(2) Men who conversed with the presumed CSW and/or subsequently walked toward nearby hotels accompanying the CSW were classified as involved males. When a target behavior occurred within the observation period, the position was recorded as a single data point.

(3) To reduce misclassification, these behavioral definitions (1 and 2) were predefined before data collection and strictly applied by a single trained observer. Classification consistency was maintained through daily review of judgments during the study period.

Data collection and statistical procedures

Observations were conducted over ten days between July 16 and July 27, 2024, from 6:30 to 8:00 p.m., excluding July 17 and July 20 due to missing data. The time frame of 6:30 to 8:00 p.m. was selected based on pre-study field visits, during which the presence of target behaviors was found to be most frequent in the early evening hours. The observer installed the free software GPS logging application RouteHistory version 2.9.1 (developed by Kazuhiko Oda, Japan) on an iPhone 14. After visually confirming a target, the observer verified the position in the app and added a point record [9].

All observations were performed by a single investigator, who classified targets according to predefined criteria. Inter-observer agreement could not be calculated due to the single-observer design; however, consistency was maintained by applying the same criteria daily. Four variables were collected: recording date, latitude, longitude, and apparent sex. No personal information (e.g., names, facial images, or unique identifiers) was collected. No IDs were assigned to data points, and individuals were not tracked across time points; thus, re-identification risk does not arise. The investigator wore unobtrusive clothing, and no contact with residents or observed individuals occurred.

Apparent sex was judged by visual observation and entered as an attribute (“F” for female, “M” for male) when adding the point; this categorization does not preclude gender diversity but was adopted for the analytic purpose of this study. The log file (GPX format) was transferred to a research laptop after completing all observations and stored on an encrypted solid-state drive (SSD); although no personal information was included, the file was handled with data security practices. The police intervention on July 22 was observed by the investigator. Across the 10 observation days, 96 point records were obtained (female: 67; male: 29). No records were excluded for data errors. These records represent location-time observations without individual identifiers; therefore, it is possible that the same individuals were observed more than once across or within days.

Ethics statement

This study did not involve human subjects research and contained no personally identifiable information. According to the official exemption criteria set by the institutional ethics review board, the study was determined to be exempt from ethics review.

Spatial analysis in QGIS

The free, open-source software QGIS 3.36 'Friedrichshafen' was used for visualisation and spatial processing. GPX files were imported into the WGS84 (EPSG:4326) coordinate reference system, and no reprojection was performed to maintain positional consistency [10].

H3 grid generation

H3 is an open-source hierarchical hexagonal indexing system developed by Uber. Each hexagonal cell contains seven child cells at the next finer resolution, preserving parent-child relationships across resolutions and enabling multiscale analysis while maintaining spatial relatedness [11]. Because cells can be configured at variable resolutions and parent-child relationships are retained, analyses can flexibly expand to broader areas during surges or focus on smaller hotspots as needed while preserving geographic continuity-features useful for dynamic infectious disease surveillance.

In QGIS, we installed the “Density Analysis” free plugin and assigned each point to H3 grids at resolutions 13 and 14 using the plugin workflow. The h3-py library (ver. 4.3.0) (Uber Technologies, Inc., San Francisco, California, USA) was installed via the OSGeo4W Shell to support H3 operations within the environment. The mean cell areas were treated as 43.9 m² (diagonal 7.6 m) for resolution 13 and 6.3 m² (diagonal 2.7 m) for resolution 14, with the former used to overview distribution across the study block and the latter used to identify micro-clusters near the central intersection [12].

Daily layers were generated by filtering the 10-day dataset by date, and cell counts per day were computed. For cartography, counts were classified into one to four classes according to H3 resolution using Jenks' natural breaks, and symbolized on a light-yellow (class 1) to dark-red (class 4) continuous color scale. Sex-stratified maps were created by splitting the full-period data into female and male subsets, aggregating counts to resolutions 13 and 14, and applying the same symbology.

Statistical analysis

Because an incidental police intervention occurred during the observation window, we compared counts before (July 16-21, 2024; six days) and after (July 22-27; six days) the intervention using a two-sided Mann-Whitney U test. Statistical significance was set at p < 0.05 (two-sided). Medians and interquartile ranges (IQRs) were reported as descriptive statistics. The Mann-Whitney U test was performed using the wilcox.test() function in the base R stats package (ver. 4.5.0) (R Foundation for Statistical Computing, Vienna, Austria) [13].

## Results

**Figure 2:**
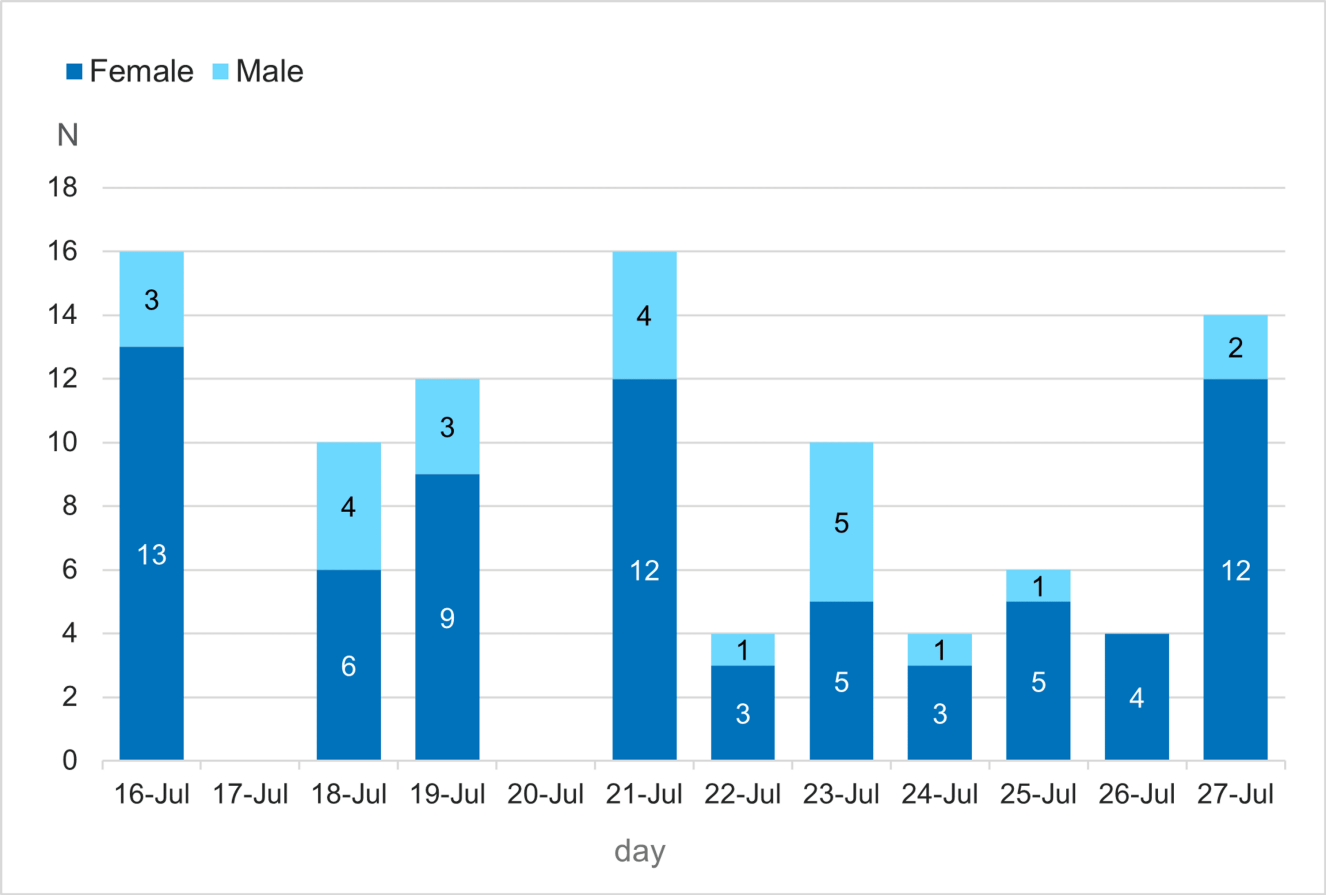
Daily counts by sex during the observation period

Figure 2 presents daily counts of observed females and males during the study period. The total number of observations was 96, with females shown in navy and males in cyan; the x-axis spans the full observation window from July 16 to July 27 (with missing data on July 17 and July 20). Females outnumbered males on all observed days; in particular, the female count was approximately fourfold the male count on July 16 and sixfold on July 27. A marked decline in counts was observed on July 22, coinciding with a police intervention within the study area.

As summarized in Table 1, we compared the median daily counts before and after the intervention. The median [IQR] daily count was 14 [11.5-16] during the six days before the intervention (July 16-21) and 4 [4-6] during the five days after the intervention (July 22-26). This reduction was statistically significant by the two-sided Mann-Whitney U test (p = 0.0095), corresponding to an approximate 70% decrease. However, when July 27 was included in the post-intervention group, the difference no longer reached statistical significance (p = 0.0506), suggesting that the effect of the intervention was short-term and transient.

**Table 1:** Change in counts before and after police intervention

Period	Days observed (n)	Median daily count [IQR]
Pre-intervention (Jul 16–21, 2024)	6	14 [11.5–16]
Post-intervention (Jul 22–26, 2024)	5	4 [4–6]
		Man-Whitney U =19.5, p<0.05

**Figure 3:**
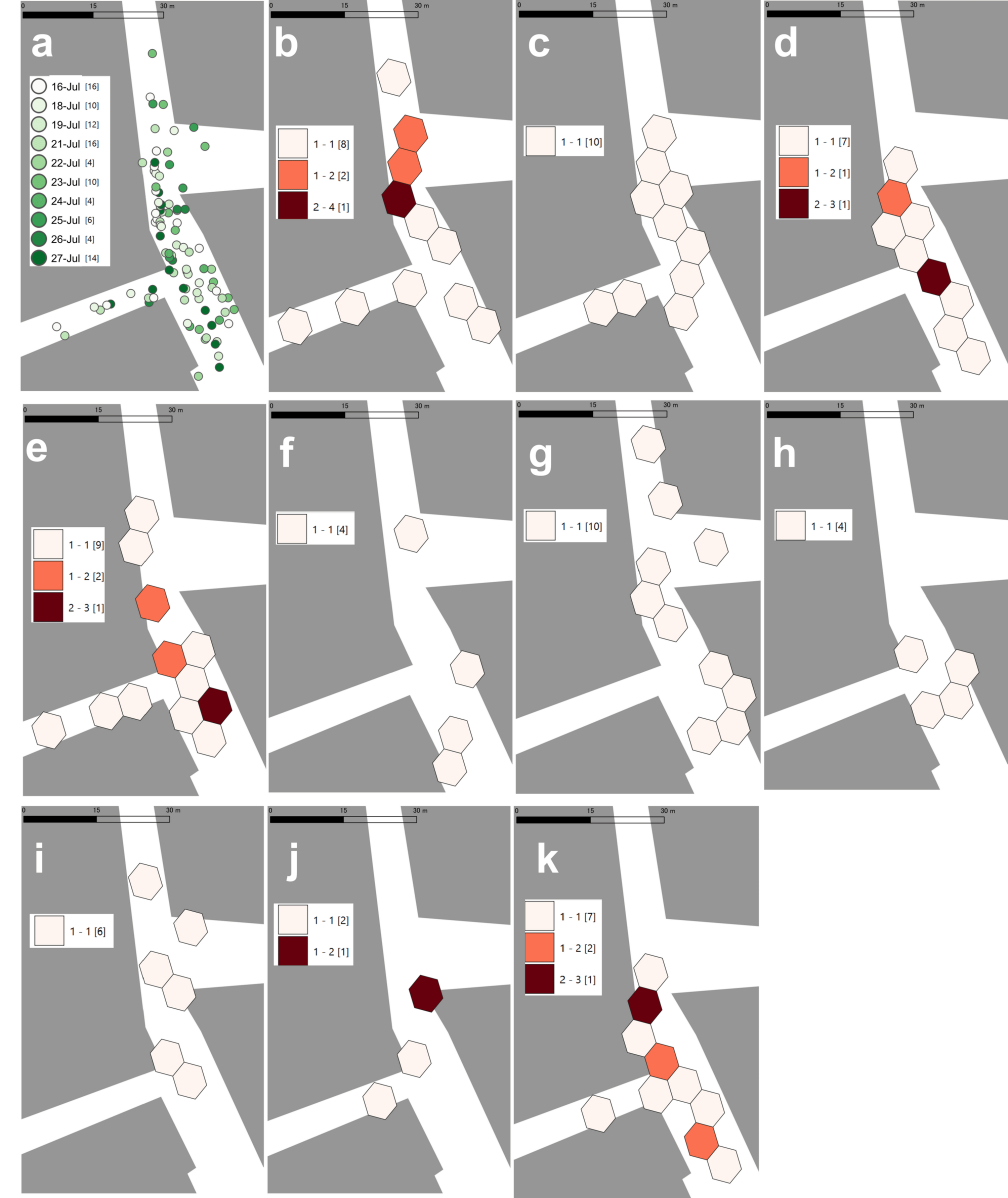
H3 cells by observation day Panel a shows all point data across the observation period. Panels b–k display H3 grids (resolution 13) generated from daily point data: b = Jul 16, c = Jul 18, d = Jul 19, e = Jul 21, f = Jul 22, g = Jul 23, h = Jul 24, i = Jul 25, j = Jul 26, k = Jul 27. © OpenStreetMap contributors.

Figure 3a maps all point locations across the entire observation period. Figures 3b-3k display H3 cells (resolution 13) for each observation day. Because Figure 3a uses a cumulative color ramp in which greener shades indicate later observation dates, it allows recognition of locations visited over the full period but makes daily clustering difficult to discern. Therefore, in Figures 3b-3k, we generated daily H3 grids at a resolution of 13 and classified cell counts so that higher counts appear in darker red. From Figure 3b through 3e (July 16-21; pre-intervention), observations were fairly widespread across the block, with clustering centered around the mid-section of the north-south street; the maximum was four observations per cell.

Post-intervention (Figure 3f-3j), the distribution differed from the pre-intervention pattern, as shown in Figure 3f-3i shows non-contiguous cells dispersed across the area, predominantly with one individual per cell. In Figure 3j (four days after the intervention), the total remained low, but cells with two observations appeared. In Figure 3k (five days after the intervention), clusters re-emerged along the mid-to-lower portion of the north-south street, with up to three observations per cell, resembling the pre-intervention pattern.

**Figure 4:**
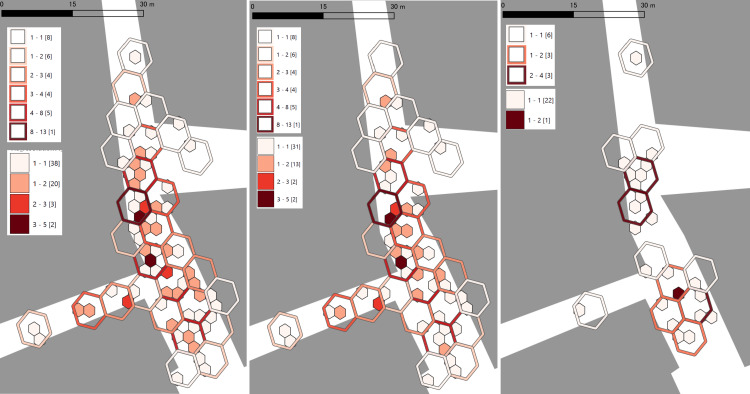
Overlay of H3 cells at two resolutions for all observations and by sex Left: all observations; center: females; right: males. © OpenStreetMap contributors.

Figure 4 overlays H3 grids created for all observations and for each sex, combining resolutions 13 and 14. In both resolutions, darker red indicates higher counts. Because a smaller numeric resolution denotes larger cells, the filled small cells correspond to resolution 14 and the colored outlines to resolution 13. Resolution-14 cells nest within their resolution-13 parent cells, and their counts correspond via the parent-child relationship. Given the relatively larger number of observations among females and in the combined dataset, cells with ≥2 observations appear even at resolution 14, enabling the identification of localized clustering.

In terms of clustering characteristics, the combined dataset (left panel) shows multiple-person cells concentrated from the mid-section toward the lower portion of the north-south street. The female-only grid (center panel) exhibits a similar pattern to the combined dataset, with a tendency for adjacent cells to form continuous clusters more frequently than in the male-only grid. In contrast, the male-only grid (right panel) at resolution 14 is dominated by single-person cells due to the smaller sample size, making clustering less apparent. However, the distribution suggests two focal areas (mid-street and lower street), with men spaced more apart. When aggregating to the coarser resolution, 13-preserving the parent-child structure-cells with ≥2 observations emerge, confirming two cluster locations.

## Discussion

Key findings

This study demonstrated that an approach combining H3 and QGIS-independent of administrative boundaries-can characterize short-term dynamics of risk behaviors and responses to external events in geographic space, supporting public health surveillance.

Significance of the H3+QGIS approach

Conventional STD surveillance is often aggregated by health-center jurisdictions or postal codes, which can obscure localized clustering of risk behaviors. The H3 index, developed by Uber, hierarchically subdivides hexagonal cells, preserving relationships between parent and child cells and enabling multiscale analysis without reliance on administrative units [3,4,11]. In this study, resolution 14 (6.3 m²) captured intersection-scale hotspots, while resolution 13 (43.9 m²) revealed block-level trends; notably, the approach captured a temporal reduction in activity coinciding with an external event (police intervention). However, due to the small sample size and observational nature of the study, this reduction should be interpreted as a temporal association rather than a causal effect.

In prior work on respiratory infections, H3-based methods improved the precision of COVID-19 contact tracing and enabled fine-grained epidemic reconstruction in mobility simulations [7,6]. Applications to STDs, however, have been limited. Our findings indicate that high-resolution, time-series monitoring of STD-related risk behaviors is feasible while preserving privacy, offering a potential foundation for spatial targeting in practice. Because cell-aggregated data can be shared without handling individual coordinates, interagency collaboration becomes more tractable with reduced ethical and legal barriers.

Public health implications

The high-resolution risk maps generated here can help public health nurses prioritize where and when to deploy limited resources. For example, clear clustering of presumed CSWs and involved males near the intersection suggests that targeted outreach-such as on-site education materials or mobile STD testing-could suppress risk behaviors over short periods. Micro-hotspots detected at resolution 14 may be missed by administrative-unit analyses yet can directly inform localized interventions (e.g., peer education, condom distribution, referral to health-center testing).

Beyond STDs and respiratory infections, similar methods may support dengue risk prediction and preparedness planning [14]. In Japan, public health nurses are responsible for a wide range of notifiable diseases under the Infectious Diseases Control Law, from viral hemorrhagic fevers to tuberculosis, measles, severe fever with thrombocytopenia syndrome (SFTS), and STDs [15]. Operationalizing H3 and QGIS within a One Health-oriented emergency management framework could facilitate efficient, timely, and evidence-based activities. Furthermore, because H3 grids allow sharing of cell-level aggregates, public health centers can coordinate with the Ministry of Health, Labour and Welfare, and other agencies or NGOs without exchanging personally identifiable information advantage in STD-related collaboration. Experience from COVID-19 suggests that combining privacy protection with high-resolution analytics can accelerate epidemiologic responses [16]; similar benefits may be achievable with this approach.

Limitations

This study has several limitations that constrain the interpretation and generalizability of the findings. First, the data were collected from a single urban area over 10 days, with a total of 96 observations. Seasonal patterns, other geographic contexts, and long-term trends were not captured. Therefore, the findings should be interpreted as preliminary and exploratory, and broader applicability remains uncertain.

Second, the classification of presumed commercial sex workers (CSWs) and involved males was based solely on visual behavioral observation without direct interviews or biological confirmation. Although predefined operational criteria were consistently applied by a trained observer and reviewed daily, the potential for misclassification remains, such as including passersby who briefly stopped.

Third, all observations were conducted by a single investigator. While consistent application of criteria was maintained, the lack of inter-observer assessment limits evaluation of internal validity, and observer bias cannot be entirely excluded.

Fourth, only behavioral indicators were analyzed. No biological data (e.g., STD diagnosis) or other risk factors-such as number of partners, condom use, or substance use-were assessed. As a result, observed behaviors cannot be directly linked to infection risk or underlying causes.

Fifth, while a statistically significant reduction was observed after the police intervention (July 22-26), this did not remain significant when including July 27. Thus, causal inference should be made cautiously.

Despite these limitations, the study provides initial support for the feasibility of high-resolution H3-based surveillance of STD-related behaviors. Future research should incorporate longer durations, multiple sites, biological linkage, and additional risk factor data to validate and expand upon these findings.

Future directions

To embed this H3-QGIS approach in routine public health practice, observation periods and sites should be expanded, and time-dependent factors (e.g., seasonality, holidays, local events) should be incorporated. Applying a common protocol across multiple cities would permit comparisons by population size and urban form and improve external validity. Re-aggregating anonymized STD case data to H3 cells and integrating them with behavioral indicators would allow testing associations while accounting for spatial autocorrelation. Reliability could be improved by deploying multiple observers, assessing inter-rater agreement, and complementing human observation with imaging analytics or Bluetooth-based sensors.

Technically, a real-time dashboard that automatically updates cell-level aggregates could enable public health nurses to review emerging hotspots from the office and time outreach accordingly. Systematic progress along these lines may establish the H3-QGIS method as a core tool for STD surveillance and further strengthen the evidence base for public health policy.

## Conclusions

Using hierarchical hexagonal grids (H3) with QGIS, we captured fine-scale clustering and short-term fluctuations of STD-related risk behaviors in an urban micro-area, including a transient decline following an external event. These observations reflect behavioral indicators at specific location-time points, not unique individuals or confirmed STD cases; therefore, no causal inference can be drawn. Because analyses are independent of administrative boundaries and use aggregated, non-identifying data, this approach appears feasible for monitoring risk behaviors in a privacy-conscious manner and may help public health nurses target outreach by place and time. However, limitations such as reliance on a single observer, small sample size, and short duration suggest that broader validation across cities and seasons, with anonymized case data and inter-observer reliability checks-would strengthen future applications.
